# 荧光法在单孔胸腔镜解剖性肺段切除术中识别段间交界线的可行性研究

**DOI:** 10.3779/j.issn.1009-3419.2021.102.38

**Published:** 2021-11-20

**Authors:** 云刚 孙, 强 张, 朝 王, 丰 邵

**Affiliations:** 1 210029 南京，南京医科大学附属脑科医院（胸科院区）胸外科 Department of Thoracic Surgery, Affiliated Nanjing Brain Hospital, Nanjing Medical University, Nanjing Chest Hospital, Nanjing 210029, China; 2 210029 南京，南京医科大学肺部结节诊疗中心 Pulmonary Nodule Diagnosis and Treatment Research Center, Nanjing Medical University, Nanjing 210029, China

**Keywords:** 单孔胸腔镜, 解剖性肺段切除术, 段间交界线, 荧光法, 改良膨胀萎陷法, Uniportal thoracoscopy, Segmentectomy, Intersegmental boundary line, Fluorescence method, Modified inflation-deflation

## Abstract

**背景与目的:**

肺段切除术逐渐成为影像学上表现为早期肺癌的肺部小结节的标准手术方式之一。本研究通过对比单孔胸腔镜下荧光法与改良膨胀萎陷法在界定段间交界线的围手术期结果，评估荧光法应用于肺段切除术的有效性和可行性。

**方法:**

回顾性分析2018年2月-2020年8月期间在南京胸科医院胸外科接受单孔胸腔镜肺段切除术的连续198例患者的围手术期数据。在三维智能交互式定性和定量分析（intelligent/interactive qualitative and quantitative analysis-three dimensional, IQQA-3D）图像分析系统的指导下，精确识别和解剖离断靶段结构，继而通过荧光法或改良膨胀萎陷法确认段间交界线。评价两种方法的临床疗效和术后并发症。

**结果:**

荧光法组有98%的患者呈现出清晰的段间交界线，甚至部分患者使用了较低剂量的吲哚菁绿（indocyanine green, ICG）。相比改良膨胀萎陷法，荧光法的段间交界线的清晰呈现时间[(23.59±4.47) s *vs* (1, 026.80±318.34) s, *P* < 0.01]和手术时间[(89.3±31.6) min *vs* (112.9±33.3) min, *P* < 0.01]明显缩短。改良膨胀萎陷法术后长时间漏气的发生率高于荧光法组（8.0% *vs* 26.5%, *P*=0.025）。两组的术中失血量、术后胸管持续引流时间、术后住院时间、手术切缘宽度及其他术后并发症均无明显差异（*P*均 > 0.05）。

**结论:**

荧光法可以高度准确地识别段间交界线，使得解剖性肺段切除术更加简单、更加快速，因此荧光法有可能成为一种可行且有效的技术，以提高单孔胸腔镜肺段切除术的质量。

## Abstract (en)

**Background and objective:**

Segmentectomy has gradually become one of the standard surgical methods for small pulmonary nodules with early lung cancer on imaging. This study aimed to investigate the perioperative outcomes of patients who underwent uniport video-assisted thoracoscopic surgery (VATS) segmentectomy for identifying the intersegmental boundary line (IBL) by the near-infrared fluorescence imaging with intravenous indocyanine green (ICG) method or the modified inflation-deflation (MID) method and assess the feasibility and effectiveness of the ICG fluorescence (ICGF)-based method.

**Methods:**

We retrospectively analyzed the perioperative data in total 198 consecutive patients who underwent uniport VATS segmentectomy between February 2018 and August 2020. With the guidance of preoperative intelligent/interactive qualitative and quantitative analysis-three dimensional (IQQA-3D), the targeted segment structures could be precisely identified and dissected, and then the IBL was confirmed by ICGF-based method or MID method. Clinical effectiveness and postoperative complications of the two methods were evaluated.

**Results:**

An IBL was visible in 98% of patients by the ICGF-based group, even with the low-doses of ICG. The ICGF-based group was significantly associated with the shorter IBL clear presentation time [(23.59±4.47) s *vs* (1, 026.80±318.34) s] (*P* < 0.01) and operative time [(89.3±31.6) min *vs* (112.9±33.3) min] (*P* < 0.01), compared to the MID group. The incidence of postoperative prolonged air leaks was higher in the MID group than in the ICGF-based group (8.0% *vs* 26.5%, *P*=0.025). There were no significant differences in bleeding volume, chest tube duration, postoperative hospital stays, surgical margin width and other postoperative complications (*P* > 0.05).

**Conclusion:**

The ICGF-based method could highly accurately identify the IBL and make anatomical segmentectomy easier and faster, and therefore has the potential to be a feasible and effective technique to facilitate the quality of uniport VATS segmentectomy.

肺癌已成为全球发病率和死亡率最高的恶性肿瘤，是严重威胁人类生命的疾病^[1]^。随着高分辨率胸部计算机断层扫描（computed tomography, CT）的发展和近年来体检的普及，越来越多的肺结节被检测出来，其中一部分高度怀疑是癌前病变或早期肺癌，需要进行手术切除^[2, 3]^。单孔胸腔镜（uniport video-assisted thoracoscopic surgery, UVATS）解剖性肺段切除术，遵循最大程度保留肺功能的原则同时满足肿瘤学的要求，已成为小结节型早期非小细胞肺癌（non-small cell lung cancer, NSCLC）的可靠手术方式之一^[4-6]^。

此外，有研究^[7, 8]^报道了解剖性肺段切除术因保留术后肺功能的优势，大大提高了患者的生活质量。然而，与标准肺叶切除术相比，肺段切除术被认为是一个更复杂的手术，尤其是完全通过单孔胸腔镜方法进行手术操作时，因为肺段靶段结构容易出现个体差异，并且肺段面之间的交界线（intersegmental boundary line, IBL）的精准识别在技术要求方面也极具挑战性。尽管之前报道了几种识别段间交界线的方法，可协助胸外科医生在肺段切除术过程中识别段间交界线，但是主要有两种方法可以呈现精准的段间交界线：使用改良膨胀萎陷法^[9-11]^或近红外荧光成像联合静脉注射吲哚菁绿（indocyanine green, ICG）^[12-14]^。在单孔胸腔镜操作背景下，由于改良膨胀萎陷法需要在手术过程中使得肺组织完全膨胀，这会使手术视野变窄并导致手术操作空间不足，而静脉注射ICG的荧光法因无需肺再充气膨胀，对主刀医生手术视野干扰较小，被认为更有利于识别段间交界线。在之前的研究中，我们已经证明荧光法以肺动脉循环为基础，其确立的段间交界线，与改良膨胀萎陷法高度一致^[15]^。然而，据我们所知，目前缺乏回顾性研究或前瞻性试验，评估这两种方法在单孔胸腔镜肺段切除术中的围手术期结果。因此，我们回顾性分析了南京市胸科医院通过该两种方法在单孔胸腔镜肺段切除术中确定段间交界线患者的围手术期结果，并评估荧光法的有效性和可行性。

## 1 资料与方法

### 1.1 基本资料

本研究回顾性分析了从2018年2月-2020年8月连续198例在南京市胸科医院胸外科行单孔胸腔镜肺段切除术患者的临床资料，这些患者采用改良膨胀萎陷法或荧光法确定段间交界线。纳入标准：①根据美国国家综合癌症网络（National Comprehensive Cancer Network, NCCN）指南，肺结节符合肺段切除术的指征；②心肺功能检查正常；③术前与患者沟通单孔胸腔镜肺段切除术的的手术风险和益处后，获得知情同意。排除标准：①严重的胸膜粘连；②有碘剂或ICG过敏史；③肺部多发结节且不在同一肺段内。本研究经医院伦理委员会批准（批准号：2020-KY093-01）。

### 1.2 术前三维重建模拟

对拟行肺段切除术的患者术前常规检查胸部增强CT，然后利用三维智能交互式定性和定量分析（intelligent/interactive qualitative and quantitative analysis-three dimensional, IQQA-3D）系统对胸部增强CT的数据进行术前重建三维模型，定位肺结节所在靶段，识别出靶段的主要结构。以疑似恶性的结节为中心，结合结节至段间静脉的距离，保证足够的手术切除范围≥2 cm或肿瘤的最大直径，然后对重建的动态三维图像进行靶段结构的模拟离段。此外，段间静脉是一种有助于识别段间交界线的标记，在肺段切除术时应予以保留。

### 1.3 手术操作

所有患者经全麻双腔气管插管，健侧单肺通气，侧卧位，在腋前线第4或第5肋间通过长度约3 cm的切口进行单孔胸腔镜解剖肺段切除术。手术过程中，在胸腔镜屏幕前放置另一台计算机以实时显示重建的三维图像，指导术中肺段结构的观察，实现精确术中导航。离断靶段的肺动脉和段内静脉一般采用4号缝线缝扎或超声刀切断，离断靶段的支气管采用直线切割缝合器切断。在荧光法中，在处理完靶段结构后，抽取5 mg剂量的ICG（辽宁济世制药有限公司，H20045514）经患者外周静脉快速推注，并将红外荧光胸腔镜（PINPOINT荧光系统）调成荧光模式观察肺组织染色区域的改变。可以在荧光模式看到需要保留的肺组织以“染色成绿色”显示，而需切除的靶段肺组织则未被染色。因此，通过明显的颜色区域的差异可以呈现出清晰的段间交界线，然后用电凝棒在脏层胸膜上进行标记（图 1）。术中如有需要，可多次注射ICG，本研究中每一个体的最大剂量为25 mg。在改良膨胀萎陷法中：在精准离断靶段结构后，嘱咐麻醉医师在控制气道压力20 cmH_2_O的情况下，经双肺通气，术侧用纯氧使肺组织完全膨胀，然后恢复健侧单肺通气，靶段因肺循环血流中断保持膨胀状态，而保留的肺组织将完全萎陷。根据膨胀与萎陷的界限对比，同样可以在胸膜上出现不规则弯曲的过渡带，形成清晰的段间交界线，并用电凝棒标记（图 2）。为适形裁剪段间平面，我们一般使用超声手术刀或电凝勾沿着胸膜标记，结合段间静脉走行，将肺门组织结构剥离至段间实质外1/3，而后根据胸膜标记，使用直线切割缝合器对外周的段间平面进行裁剪。

**图 1:**
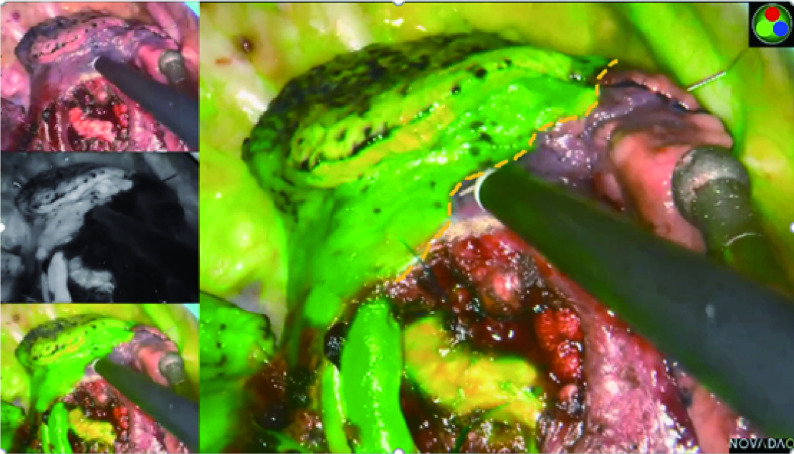
使用PINPOINT荧光成像系统显示，通过静脉注射吲哚菁绿法的近红外荧光成像清楚地识别了段间交界线。 The intersegmental boundary line was clearly identified via the near-infrared fluorescence imaging with intravenous indocyanine green method using PINPOINT endoscopic fluorescence imaging system.

**图 2:**
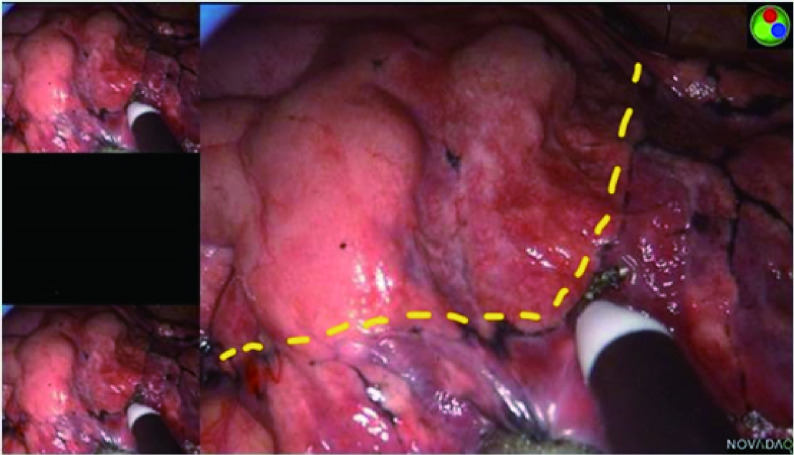
使用PINPOINT荧光成像系统显示，通过改良膨胀萎陷法清晰地识别了段间交界线。 The intersegmental boundary line was clearly identified via the modified inflation-deflation method using PINPOINT endoscopic fluorescence imaging system.

根据术中冷冻病理诊断结果，恶性肿瘤患者应行肺门和纵隔淋巴结清扫或采样。然后进行肺漏气测试，如果术中发现肺断面有少量漏气，可用奈维或纤维蛋白胶覆盖创面，如果发现是靶段支气管残端明显漏气，则需3-0 prolene缝合加固。最后在单孔手术切口处置入22 F胸腔引流管，关闭胸腔。术后在确认肺复张良好、无漏气、胸腔引流量24 h小于200 mL的情况下予以拔除。另外在本研究中，患者术后的病理分期是基于第8版的肿瘤原发灶-淋巴结-转移（tumor-node-metastasis, TNM）分期系统。

### 1.4 观察指标

本研究纳入两组患者的基本临床资料和围手术期数据。围手术期数据包括手术时间、术中失血量、术后胸管持续引流时间、术后住院时间、段间交界线识别率、段间交界线呈现时间、手术切缘宽度和手术相关并发症。与手术相关的术后并发症主要包括肺部感染、肺不张、咯血和术后持续漏气（> 7 d）。

手术时间计时方法：从切皮开始到皮肤缝合完成。

在荧光法中，段间交界线呈现时间计时方法：从经外周静脉注入ICG到呈现出明显的段间交界线颜色差。

在改良膨胀萎陷法中，段间交界线呈现时间计时方法：从全肺完全膨胀到出现一清晰且不再移位的膨胀萎陷交界线。

### 1.5 统计学分析

采用SPSS 22.0软件进行统计学分析。连续数据以均数±标准差（Mean±SD）表示，组间差异比较采用独立样本*t*检验。分类变量以频率和百分比（%）表示，组间差异比较采用统计学方法*χ*^2^或*Fisher*精确检验。*P* < 0.05为差异具有统计学意义。

## 2 结果

### 2.1 两组基本临床资料

198例连续行单孔胸腔镜肺段切除术的患者中，100例采用基于静脉注射ICG的荧光法，98例采用改良膨胀萎陷法进行段间交界线的识别。患者的年龄、性别、吸烟状况、肺功能、合并症、肿瘤大小、胸膜粘连和病理诊断的结果汇总于表 1，各组间观察变量无明显差异（*P*均 > 0.05）。两组肺段切除部位资料见表 2。

**表 1:** 两组患者基本临床资料 Clinical characteristics of patients of two groups

Characteristics	ICGF-based group (*n*=100)	MID group (*n*=98)	*P*
Age (Mean±SD, yr)	59.8±10.5	60.6±7.9	0.500
Gender [*n* (%)]			0.242
Male	43 (43.0%)	48 (49.0%)	
Female	57 (57.0%)	50 (51.0%)	
Smoking status [*n* (%)]			0.402
Ever	80 (80.0%)	76 (77.6%)	
Never	20 (20.0%)	22 (22.4%)	
FEV_1_ (Mean±SD, L)	2.5±0.6	2.4±0.7	0.789
COPD [*n* (%)]			0.391
Yes	10 (10.0%)	12 (12.2%)	
No	90 (90.0%)	86 (87.8%)	
Tumor size (Mean±SD, mm)	11.6±3.9	11.6±3.7	0.893
Pleural adhesions [*n* (%)]			0.439
Yes	86 (86.0%)	12 (12.2%)	
No	14 (14.0%)	86 (87.8%)	
Pathological diagnosis [*n* (%)]			0.579
AAH	4 (4.0%)	1 (1.0%)	
AIS	34 (34.0%)	37 (37.8%)	
MIA	52 (52.0%)	51 (52.0%)	
IAC	10 (10.0%)	9 (9.2%)	
SD: standard deviation; FEV_1_: forced expiratory volume in one second; COPD: chronic obstructive pulmonary disease; AAH: atypical adenomatous hyperplasia; AIS: adenocarcinoma *in situ*; MIA: minimally invasive adenocarcinoma; IAC: invasive adenocarcinoma.			

**表 2:** 两组患者的肺段分布 Position of target segments in two groups

Segmentectomy position	ICGF-based group (*n*=100)	MID group (*n*=98)
Right lobe		
Single segment		
S^1^	10 (10.0%)	13 (13.3%)
S^2^	14 (14.0%)	4 (4.1%)
S3	7 (7.0%)	19 (19.4%)
S^6^	7 (7.0%)	4 (4.1%)
S^8^	3 (3.0%)	1 (1.0%)
Subsegment		
S^8a^	3 (3.0%)	2 (2.0%)
Combined segmentectomy		
S^1+2^	0 (0.0%)	2 (2.0%)
S^2b + S3a^	4 (4.0%)	2 (2.0%)
S^7+8^	3 (3.0%)	5 (5.1%)
S^9+10^	3 (3.0%)	3 (3.1%)
Left lobe		
Single segment		
S^3^	7 (7.0%)	6 (6.1%)
S^6^	8 (8.0%)	6 (6.1%)
S^8^	3 (3.0%)	1 (1.0%)
Subsegment		
S^8a^	2 (2.0%)	1 (1.0%)
Combined segmentectomy		
S^1+2^	5 (5.0%)	9 (9.2%)
S^1+2+3^	7 (7.0%)	7 (7.1%)
S^4+5^	5 (5.0%)	7 (7.1%)
S^7+8^	7 (7.0%)	6 (6.1%)
S^9+10^	2 (2.0%)	0 (0.0%)

### 2.2 两组围手术期结果

荧光法组的所有患者均未发生与静脉注射ICG相关的不良事件，两组均未发生围手术期30天内死亡或再入院事件。两组围手术期结果总结在表 3中，包括手术时间、术中失血量、术后胸管持续引流时间、术后住院时间、段间交界线识别率、段间交界线呈现时间、手术切缘宽度和手术相关并发症。

**表 3:** 两组患者的围手术期结果 Perioperative data of two groups

Variable	ICGF-based group (*n*=100)	MID group (*n*=98)	*P*
Operative time (Mean±SD, min)	89.3±31.6	112.9±33.3	< 0.010
Bleeding volume (Mean±SD, mL)	57.2±41.7	67.4±56.2	0.146
Presentation time (Mean±SD, s)	23.6±4.4	1, 027.7±322.5	< 0.010
Identification rate	98.0%	89.8%	0.015
Surgical margin width (Mean±SD, cm)	2.5±0.5	2.4±0.5	0.199
Chest tube duration (Mean±SD, d)	4.3±1.8	4.7±2.1	0.166
Postoperative complications			
ICG-related complications	0 (0.0%)	-	
Prolonged air leaks (> 7 d)	8 (8.0%)	26 (26.5%)	0.025
Pulmonary infection	2 (2.0%)	1 (1.0%)	0.508
Hemoptysis	6 (6.0%)	9 (9.0%)	0.282
Atelectasis	4 (4.0%)	2 (2.0%)	0.351
Postoperative hospital stays (Mean±SD, d)	5.5±1.7	5.9±2.1	0.184
ICG: indocyanine green.			

荧光法组有98%的患者呈现出清晰的段间交界线，甚至部分患者使用较低剂量的ICG，但是2例右侧S^1^的患者合并有严重肺气肿未显示较为明显的段间交界线。改良膨胀萎陷法组有89.8%的患者呈现出清晰的段间交界线，另有10例患者经过较长时间的等待后仍未出现明显的段间交界线，包括4例右侧S^1^、2例S^3^、3例S^6^和1例左侧S^1+2^，其中有6例患者合并有肺气肿，4例患者合并有胸膜粘连。相比改良膨胀萎陷法，荧光法的段间交界线的清晰呈现时间[(23.59±4.47) s *vs* (1, 026.80±318.34) s, *P* < 0.01]和手术时间[(89.3±31.6) min *vs* (112.9±33.3) min, *P* < 0.01]明显缩短。在三维重建图像的引导下，荧光法组与改良膨胀萎陷法组手术切缘宽度无明显差异[(2.5±0.5) cm *vs* (2.4±0.5) cm, *P*=0.199]，两组均达到了足够的手术切除范围，均符合≥2 cm或结节最大直径的肿瘤学要求。荧光法组相对改良膨胀萎陷法有较少的术中失血量、较短的术后胸管持续引流时间和术后住院时间，但各组间差异无统计学意义（*P*均 > 0.05）。

表 3也记录了两组患者的手术相关并发症。改良膨胀萎陷法的术后长时间漏气的发生率高于荧光法组[8.0% (8/100) *vs* 26.5% (26/98), *P*=0.025]。26例（13.1%）患者出现术后持续性漏气，其中荧光法组8例，改良膨胀萎陷法组18例；肺部感染3例（1.4%），其中荧光法组2例，改良膨胀萎陷法组1例；咯血15例，其中荧光法组6例，改良膨胀萎陷法组9例；肺不张6例，其中荧光法组4例，改良膨胀萎陷法组2例；术后肺部感染、肺不张、咯血等并发症两组比较无明显差异（*P*均 > 0.05），术后出现持续性漏气等并发症的患者经保守治疗后均痊愈，无再次手术病例。

## 3 讨论

近年来，一些回顾性研究^[16-19]^表明胸腔镜解剖性肺段切除术是治疗早期肺癌的标准手术方式之一，尤其是当肿瘤直径 < 2 cm时，患者的预后与肺叶切除术相似。然而，即使是经验丰富的胸外科医师，胸腔镜解剖性肺段切除术也是需要克服许多困难和局限性的一项苛刻技术，尤其是采用单孔胸腔镜入路时。因此，由于肺段解剖结构的复杂性和较高段间交界线识别的技术要求，胸腔镜下肺段切除术仍然是一个重大挑战。

对于肺段切除术，识别段间交界线是术中的一个关键步骤，它直接关系到足够的手术切缘宽度和较少的术后并发症。肺段切除术中如不能准确识别段间交界线，可能导致手术切除肿瘤边缘的距离不够、靶段或病变残留、肺组织的大范围过度切除甚至中转为肺叶切除、医源性段间静脉损伤、术后并发症如肺部感染、肺不张、咯血、术后长时间漏气甚至再次插入胸管等。因此，基于重建的动态三维图像对靶段结构进行精确的解剖是获得清晰的段间交界线的前提。在我们中心，常规使用IQQA系统进行靶段动脉、静脉和支气管的3D重建图像，以便于术前模拟肺段切除术^[15]^。此外，重建的动态三维图像能准确识别解剖变异特征，显著降低术后并发症的发生率，另外根据经验识别靶段结构也是必要的^[20]^。尽管既往研究报道了许多识别段间交界线的方法，但是主要有两种方法可以呈现精准的段间交界线：使用改良膨胀萎陷法^[9-11]^或近红外荧光成像联合静脉注射ICG法^[12-14]^。

基于此研究大量的临床数据，我们发现荧光法对段间交界线的识别率与全球公认的传统改良膨胀萎陷法相当。此外，在之前的研究中，我们已经证明荧光法创建的段间交界线与改良膨胀萎陷法显示的高度一致，与真实段间交界线完全吻合^[15]^。对于肺功能受损的患者，如肺气肿或广泛的胸膜粘连，由于肺顺应性降低，使用改良膨胀萎陷法很难准确地识别段间交界线^[9]^。在本研究中，改良膨胀萎陷法无法清晰显示出10例患者的段间交界线，所有这些患者都有肺气肿或胸膜粘连。然而，仅2例患者的段间交界线无法通过基于肺循环血流量的荧光法明确识别，且该2例患者均存在严重的肺气肿。肺气肿的肺循环血流量远低于正常肺的肺循环血流量，因此在肺循环血流量极度减少的情况下，可能难以通过荧光法清楚地显示肺气肿患者的段间交界线^[21]^。但是，在其他肺气肿患者中通过荧光法可以清晰呈现出段间交界线，因此需要进一步研究来解释这个问题。

本研究显示，荧光法组段间交界线的平均显现时间较改良膨胀萎陷法组明显缩短，理所当然，荧光法组的手术时间也短于改良膨胀萎陷法组，平均两组手术时间相差约23 min。我们认为，荧光法缩短手术时间可能是由于良好的手术视野和较快呈现的清晰段间交界线，因为基于肺循环血流量的荧光法，不需要肺再膨胀。此外，在改良膨胀萎陷法中，需要经验非常丰富的麻醉师高度参与，这也不受主刀医师的能力控制。而且，肺再完全膨胀阻挡干扰了手术视野，导致在单孔胸腔镜的背景下手术操作空间不足，增加了手术难度，尤其是肺气肿患者，可能需要更长的时间进行肺萎陷，段间交界线不能较清晰显示。还有一点，再膨胀的肺实质不易从较小的单孔切口处取出，标本容易碎裂，导致病灶触诊困难，可能进一步延长切口，加重了患者术后胸部疼痛。从理论上讲，手术时间缩短可以显示出更好的围手术期效果，加速患者的康复。本研究发现，两组患者的围手术期并发症、术后长时间肺部漏气（诊断标准：漏气量 > 50 mL/min，持续时间超过7 d）存在较为明显的统计学差异。在改良膨胀萎陷法组中有较高比例发生术后长时间漏气，尤其是在段间交界线不能清晰显示的患者中，这可能是由于段间交界线显示不清楚，需要使用超声刀或电凝勾将段门完全打开，以沿段间静脉走行判断段间交界线，而段间静脉通常位于肺组织深部，切开更多肺段间组织，可能导致更多的肺组织破损，导致术后更高比例的漏气，尤其是肺质量本身就比较差的患者^[22, 23]^，虽然本研究在段间交界面的处理上使用奈维或纤维蛋白胶覆盖创面以减少术后肺漏气，但是仍有较高发生率，对于这一结果尚不明确是由于治疗策略差异的原因，还是与本研究样本相对较小有关，需要后续进一步研究具体分析。然而，本研究发现两组之间在其他的围手术期结果方面并没有统计学差异。尽管荧光法组显示出术中出血量减少、胸管持续时间和术后住院时间缩短的趋势，但差异没有达到统计学意义。此外，术后长时间漏气患者的胸管持续时间中位数为8.2 d。长时间的漏气肯定会延长胸管的拔除时间。这种矛盾可能与长期漏气的比例相对较小和样本量有限有关。由于重建的动态3D图像放置在操作者屏幕前进行术中实时导航，可以最大限度地减少肺段血管和支气管的意外损伤，提高清晰段间交界线的识别率。这也可能解释了本研究中两组术中出血量小、切缘均足够充分的原因。

本研究有一些局限性。首先，与其他回顾性研究一样，潜在的选择偏差无法避免。其次，PINPOINT荧光成像系统的成本相对较高，但是本中心购买的此昂贵设备对患者来说并没有经济负担，不需要额外的手术费用。在本院，荧光胸腔镜设备可以代替标准的胸腔镜由不同的手术团队进行大部分胸腔手术，因为PINPOINT荧光成像系统与标准胸腔镜系统具有大部分相同的参数规格，减少了重新购买其他胸腔镜设备的费用。第三，之前的多项研究^[14, 24]^已经证实，静脉注射吲哚菁绿是安全，可以在胸外科手术中使用。在本研究中，无论患者的体重如何，统一使用5 mg的标准剂量ICG足够呈现出清晰的段间交界线，而且没有发生ICG相关的并发症。在本研究中ICG的使用剂量与日本报道^[25, 26]^相比仍有较大差异，我们认为这可能是由于荧光设备对近红外光的识别灵敏度不同所致。我们使用了PINPOINT荧光成像系统，日本团队使用了Olympus荧光设备（Olympus Co., Ltd, Tokyo, Japan），此外，如此小剂量的ICG还提高了手术的安全性，而且没有药物毒性。第四，本研究也发现与日本团队报道的同样问题，就是荧光法显示的段间交界线染色持续时间相对较短，因此，在注射ICG后，准备好快速用电凝棒在脏层胸膜上标记段间交界线是非常重要的。由于荧光发产生的段间交界线是通过颜色差异可视化的，本研究没有发现在几分钟内使用电凝棒在脏层胸膜上标记是一种限制，而且本研究使用的ICG剂量非常小，多次注射协助段间交界线的呈现也是安全可行的。

综上所述，荧光法可以高度准确地识别段间交界线，使得解剖性肺段切除的手术流程更加简单、更加快速，相比改良膨胀萎陷法，其具有清晰呈现段间交界线的时间缩短、对手术视野干扰小、加快手术时间等显著优势，同时确保了足够的手术切缘宽度，符合肿瘤学要求，因此荧光法有可能成为一种可行且有效的技术，以提高单孔胸腔镜肺段切除术的质量，值得推广应用。
